# The Clinical Significance of PR, ER, NF-**κ**B, and TNF-**α** in Breast Cancer

**DOI:** 10.1155/2014/494581

**Published:** 2014-04-23

**Authors:** Xian-Long Zhou, Wei Fan, Gui Yang, Ming-Xia Yu

**Affiliations:** ^1^Emergency Centre, Zhongnan Hospital of Wuhan University, Wuhan, Hubei 430071, China; ^2^Department of Clinical Laboratory, Zhongnan Hospital of Wuhan University, 169 Donghu Road, Wuhan, Hubei 430071, China

## Abstract

*Objectives*. To investigate the expression of estrogen (ER), progesterone receptors (PR), nuclear factor-**κ**B (NF-**κ**B), and tumor necrosis factor-**α** (TNF-**α**) in human breast cancer (BC), and the correlation of these four parameters with clinicopathological features of BC. *Methods and Results*. We performed an immunohistochemical SABC method for the identification of ER, PR, NF-**κ**B, and TNF-**α** expression in 112 patients with primary BC. The total positive expression rate of ER, PR, NF-**κ**B, and TNF-**α** was 67%, 76%, 84%, and 94%, respectively. The expressions of ER and PR were correlated with tumor grade, TNM stage, and lymph node metastasis (*P* < 0.01, resp.), but not with age, tumor size, histological subtype, age at menarche, menopause status, number of pregnancies, number of deliveries, and family history of cancer. Expressions of ER and PR were both correlated with NF-**κ**B and TNF-**α** expression (*P* < 0.05, resp.). Moreover, there was significant correlation between ER and PR (*P* < 0.0001) as well as between NF-**κ**B and TNF-**α** expression (*P* < 0.05). *Conclusion*. PR and ER are highly expressed, with significant correlation with NF-**κ**B and TNF-**α** expression in breast cancer. The important roles of ER and PR in invasion and metastasis of breast cancer are probably associated with NF-**κ**B and TNF-**α** expression.

## 1. Introduction

Breast cancer (BC) is the most common cause of death from cancer in women and one of the important contributors to the global health burden [1]. Current routine clinical management of BC relies on few well-defined biological markers and clinicopathological variables. Although numerous molecular biomarkers have been introduced during the past decades, only few of them such as estrogen (ER) and progesterone receptors (PR) are likely to be included in routine clinical practice [2]. Currently, ER and PR levels in BC tissue have been used to predict patient's course of disease and response to adjuvant hormonal therapy [3]. Patients with tumors positive for either receptor (PR^+^/ER^+^) are generally considered hormone receptor-positive for treatment decisions.

It is accepted that the inflammation within the tumor microenvironment correlates with increased invasiveness and poor prognosis in BC [4]. Compared to normal tissues, most cytokines were overexpressed in cancer tissues, and it has been confirmed that numerous cytokines expressions were inversely correlated with the ER and PR status in BC [5]. The steroid hormone receptors PR and ER may also play important roles in the inflammatory process. Previous studies proved that PR and NF-*κ*B mutually suppress each other's activity [6]. Because of the central role of NF-*κ*B in both the inflammatory and immunological responses, inhibition of NF-*κ*B by PR may result in anti-inflammatory and immunosuppressive reaction. Previous study also confirmed PR as an anti-inflammatory agent in the endothelium, with potential for the negative regulation of immune cell trafficking into tissues [7]. In addition, ER has been identified as a regulator of the proinflammatory properties [8].

However, despite the prevailing notion that there may be more reports regarding the interactions between steroid receptors and inflammatory process to be gained, no studies have, to our knowledge, specially investigated the interrelationship of PR and ER with NF-*κ*B and TNF-*α* in BC. In the present study, we attempt to investigate the expression of PR, ER, NF-*κ*B, and TNF-*α* in human breast cancer and the possible correlations of these four biomarkers with clinicopathological features such as tumor grade, stage, and metastasis.

## 2. Methods and Materials

### 2.1. Patients

Patients pathologically diagnosed with primary breast cancer between June 2008 and June 2009 at the Department of Pathology, Zhongnan Hospital of Wuhan University (Wuhan, China), were enrolled in this study. Clinicopathological parameters including age, histological subtype, TNM stage, tumor grade, lymph node metastasis, age at menarche, menopause status, number of pregnancies, number of deliveries, and family history of cancer were evaluated. For each case, both normal breast tissue and breast cancer tissue were collected for analysis. The strept-avidin-biotin-peroxidase complex (SABC) immunohistochemical staining method was used to detect the expression of estrogen receptor (ER), progesterone receptor (PR), nuclear factor-*κ*B (NF-*κ*B), and tumor necrosis factor-*α* (TNF-*α*) in breast cancer tissues. This study has been approved by the Ethics Committee of Zhongnan Hospital of Wuhan University (Wuhan, China).

### 2.2. Immunohistochemistry

Immunohistochemical staining of the 4 *μ*m paraffin-embedded sections was performed with the SABC method using a SABC Kit (Boster Co., Wuhan, China). Briefly, 4 *μ*m sections were dewaxed in xylene (2 × 10 min) and rehydrated through an alcohol gradient: 100% ethanol (2 × 10 min), 95% ethanol (1 × 8 min), 80% ethanol (1 × 5 min), and 70% ethanol (1 × 5 min) followed by 1 × 10 min in ddH_2_O. Then, the sections were soaked in 3% hydrogen peroxide for 25 min. The sections were then incubated with rat anti-human TNF-*α*, PR, ER, or NF-*κ*B monoclonal antibody (diluted 1 : 1000, Santa Cruz Biotechnology, Santa Cruz, CA, USA) at 37°C for 1.5 h and were washed in 0.01 M PBS and exposed to secondary antibody (1 : 200, Boster Co., Wuhan, China), followed by treatment with the SABC complex, and stained with diaminobenzidine. The optical densities of the specific bands were scanned and measured by image analysis software (HPIAS 2000, Tongji Qianping Company, Wuhan, China).

### 2.3. Stained Sections Analysis

The intensity of immunostaining was assessed by two independent observers. The degree of TNF-*α*, NF-*κ*B, PR, and ER immunopositivity in tumors was graded according to Fromowitz et al. as follows [9]: pink brown staining scored 1, yellow brown scored 2, and dark brown scored 3. Positive cell rate <25% scored 1, 25–50% scored 2, 51–75% scored 3, and >75% scored 4. Then, positive cell rate score and color score were added, and the results were graded into one of three categories: score 2-3 means weakly staining; score 4-5 means moderate staining; score > 5 means strongly staining. Specimens of those categories were considered positive expression (+). Otherwise, tissue samples were considered negative (−).

### 2.4. Statistical Analysis

Data were expressed as mean ± SD, number, and percentages. The relationship between clinicopathological parameters, immunohistochemical staining intensity, and percentage of positively stained tumor cells was tested using the *χ* ^2^ or Fisher's exact tests if appropriate. Statistical analysis was performed using SPSS software (SPSS 18.0, SPSS Inc., Chicago, IL). *P* value less than 0.05 was considered significant.

## 3. Results

### 3.1. Clinicopathological Features

Clinicopathological characteristics of total 112 primary breast cancer patients are shown in Table 1. On the basis of archival pathology reports, there are 97 cases of ductal carcinoma (97/112), 6 of invasive lobular carcinoma (6/112), 4 of mucinous adenocarcinoma (4/112), 4 of intraductal carcinoma (4/112), and 1 of lobular carcinoma* in situ* (1/112). Fifty-six (50%) patients were 50 years of age or younger. All 112 patients in our study cohort were classified into different prognostic groups according to the TNM classification system. Forty-six patients had stages III-IV, while 66 patients had stages I-II. Seventy-five patients (67%) undergoing with a tumor grade I-II, while 37 patients (33%) of grade III. Lymph node metastasis was present in 60 patients (60/112, 54%). The majority (76/112, 68%) reached menarche at age of 15 years or younger, and 61 patients (61/112, 55%) in this study underwent a natural menopause. Most patients (91/112, 81%) had no family history of cancer.

### 3.2. Immunohistochemical Findings

**Figure 1:**
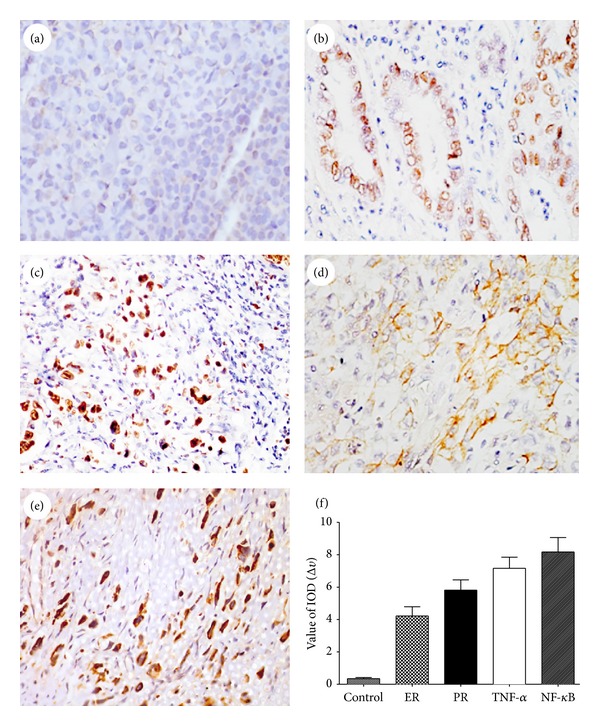
Immunohistochemical staining. (a) Control (isotype monoclonal antibody); (b) progesterone receptor (PR); (c) estrogen receptor (ER); (d) tumor necrosis factor-*α* (TNF-*α*); (e) nuclear factor-*κ*B (NF-*κ*B) (200x); (f) positive expression was also confirmed by image analysis software HPIAS 2000. IOD: integrated option density. Data were expressed as mean ± SD.

According to the immunohistochemical staining (Figure 1), ER, PR, and NF-*κ*B positive staining were limited to the nucleus, and TNF-*α* positive staining was mainly limited to the cell membranes. As seen in Table 2, the total positive expression rate of PR was 67% (75/112), while the total positive expression rate of ER was 76% (85/112) in the cancer tissue. In addition, 65% were ER^+^/PR^+^, 11% were ER^+^/PR^−^, 2% were ER^−^/PR^+^, and 22% were ER^−^/PR^−^. The total positive TNF-*α* expression rate in cancer tissue was 94% (105/112), and 84% (94/112) of cancer specimens were NF-*κ*B positive. Correspondently, the positive expression rate in the normal tissue specimens was 15% of ER, 18% of PR, 0% of NF-*κ*B, and 2% of TNF-*α*. Moreover, the positive rates of these four parameters in cancer tissue were significantly increased compared with that in the normal tissue (*P* < 0.0001, resp.).

### 3.3. The Relationship between PR, ER, NF-*κ*B, and TNF-*α*

We found a highly significant correlation between PR/ER expression and TNF-*α*/ NF-*κ*B level in breast cancer (Table 3). Sixty-two of 75 (83%) PR-positive cases were TNF-*α*-positive. In contrast, twenty-three of 37 (62%) PR-negative cases positively expressed TNF-*α*. Fourteen of 37 PR-negative cases and 13 of 75 PR-positive cases had lost TNF-*α* expression. Meanwhile, our results also showed significant correlation between ER expression and TNF-*α* levelin BC. Respectively, seventy of 85 (81%) ER-positive cases expressed TNF-*α*, while only 15 of 27 (56%) ER-negative cases showed TNF-*α* expression. Fifteen positive and 12 negative ER cases lost TNF-*α* expression. In addition, the correlation between PR/ER expression and NF-*κ*B expression was also observed. Seventy-four of 75 PR-positive (99%) and 80 of 85 ER-positive (94%) cases were NF-*κ*B positive, while only 20 of 37 PR-negative (54%) and 14 of 27 ER-negative (52%) cases were NF-*κ*B positive.

We also investigated the relationship between ER and PR expression as well as the relationship between NF-*κ*B and TNF-*α* expression. The PR expression was significantly correlated with the expression of ER in BC. As shown in Table 4, seventy-three ER-positive cases were also PR-positive. The expression of TNF-*α* was also correlated with the NF-*κ*B expression. Ninety TNF-*α* positive cases showed NF-*κ*B expression, while only 4 negative TNF-*α* cases were NF-*κ*B positive.

### 3.4. The Correlation of These Parameters with Clinicopathological Features

Table 5 showed the relationships between these four parameters and clinicopathological features of BC patients. The expressions of ER and PR were both correlated with tumor grade, TNM stage, and lymph node metastasis (*P* < 0.01, resp.), but not with age, histological subtype, tumor size, age at menarche, menopause status, number of pregnancies, number of deliveries, and family history of cancer (*P* > 0.05, resp.). The expressions of NF-*κ*B and TNF-*α* were significantly and strongly correlated with tumor size, tumor stage, and lymph node metastasis in breast cancer tissue (*P* < 0.05, resp.). In addition, NF-*κ*B was also positively correlated with tumor grade in BC (*P* < 0.01). However, neither NF-*κ*B nor TNF-*α* has correlation with the clinicopathological features including patient age, histological subtype, age at menarche, menopause, number of pregnancies, number of deliveries, and family history of cancer (*P* > 0.05, resp.).

## 4. Discussion

Progesterone receptor (PR) and estrogen receptor (ER) are the most widely studied markers in breast tissue [10]. Currently, clinicians rely on the results of PR and ER expression levels to make therapeutic decisions for BC patients. Moreover, both PR and ER expression levels in BC are used as predictive biomarkers of response to endocrine therapy [11]. In this study, we have analyzed the correlation of PR and ER with some known prognostic factors including patient age, histological subtype, TNM stages, age at menarche, menopause, number of pregnancies, number of deliveries, and family history of cancer. As reported in the literature, ER is expressed in about 70–75% of invasive breast cancer [12], and about 50% of breast cancer expresses the progesterone receptor (PR) [13]. It has been shown that the expression of PR is activated by ER [14]. Thus, PR expression commonly parallels ER expression in breast cancer [15]. However, these two receptors were present in only 15–30% of luminal epithelial cells and not elsewhere in normal human breast [16]. Our immunohistochemical analysis showed that the PR and ER positive expressions were mainly located in nucleus, and the positive expression rate of PR and ER was about 70%. In addition, there was a positive correlation between PR and ER. However, either ER or PR was rarely observed in normal breast tissues. These results in our study were in line with previous studies.

Tumor necrosis factor alpha (TNF-*α*) is a multifunctional cytokine involved in apoptosis, inflammation, and immunity [17]. TNF-*α* has been reported to be elevated in the blood serum of patients diagnosed with advanced stage BC and correlate with an increased number and size of metastatic sites [18]. The increased level of TNF-*α* was possibly linked to the activation of NF-*κ*B [19], which plays a crucial role in inflammation and carcinogenesis [20]. Previous study provided a significant correlation between TNF-*α* expression and the expression of putative TNF-*α*-inducible NF-*κ*B-related genes in human breast cancer [21]. In our study, the positive TNF-*α* expression rate was correlated with that of NF-*κ*B in BC. It has also proved that low levels of TNF-*α* in the extracellular matrix (ECM) of BC promote the growth and proliferation of tumor cells [22]. Recently, we have established that transmembrane TNF-alpha (tmTNF-*α*) monoclonal antibody (mAb) exerts effective antitumor activities in BC [23]. The interrelationships between the steroid hormone receptors and cytokines including TNF-*α* have been demonstrated in several studies. It is reported that increased endogenous TNF-*α* may promote tumor invasion via downregulating the PR expression in BC [24]. In addition, Chavey et al. [5] demonstrated that TNF-*α* was more abundant in PR-negative BC than in PR-positive ones. Moreover, TNF-*α* has an important role in regulating estrogen synthesis in malignant breast tissues [25]. ER may also inhibit TNF-*α* activation via repressing the TNF-responsive element (TNF-RE) and TNF promoter [26]. However, our results showed that either ER or PR has a positive correlation with both TNF-*α* and NF-*κ*B expression in BC. This may be associated with the different functions of two isoforms of TNF-*α*, the transmembrane, and secretory TNF-*α*. Further study would be needed to investigate the possible underlying mechanisms.

The relationship between steroid hormone receptors PR/ER and clinicopathological parameters remains uncertain. Several studies demonstrated that ER expression has strong correlation with histological subtype [27] and patient age at the time of diagnosis [28, 29], while some other studies reported that PR has no positive relationship with clinicopathological features including patient age and menopause status [30]. In our study, both PR and ER expressions were correlated with tumor grade, TNM stage, and lymph node metastasis. Moreover, expression of both TNF-*α* and NF-*κ*B was correlated with tumor size, tumor grade, and TNM stage. Those results indicate that these four parameters may be involved in the carcinogenesis of breast cancer and may play an important role in invasion and metastasis of BC. Considering the positive correlation of steroid receptors with both TNF-*α* and NF-*κ*B expression, it is possible that the biological activities of ER and PR may be associated with the expression of NF-*κ*B and TNF-*α* in human breast. However, the initial mechanism has not been elucidated yet.

In conclusion, PR and ER are highly expressed, with significant correlation with tumor grade, TNM stage, and lymph node metastasis as well as with TNF-*α* and NF-*κ*B expression in breast cancer. Thus, these two steroid receptors may be involved in the carcinogenesis of breast cancer, and their roles in invasion and metastasis of breast cancer are probably associated with the expression of NF-*κ*B and TNF-*α*. However, additional studies are required to further elucidate these relationships.

## Figures and Tables

**Table 1:** Clinicopathological features of breast cancer patients (*n* = 112).

Clinicopathologic parameters	Number of cases (%)
Age (years) (median 50, range 35–72)	
<50	56 (50)
≥50	56 (50)
Histologic subtype	
Ductal carcinoma	97 (85.8)
Invasive lobular carcinoma	6 (5.3)
Mucinous adenocarcinoma	4 (3.6)
Intraductal carcinoma	4 (3.6)
Lobular carcinoma *in situ *	1 (0.9)
TNM stages	
I-II	66 (58.9)
III-IV	46 (41.1)
Lymph node metastasis	
Yes	60 (53.6)
No	52 (46.4)
Tumor grade	
I-II	75 (67.0)
III	37 (33.0)
Tumor size	
<5 cm	77 (68.8)
≥5 cm	35 (31.2)
Age at menarche (years)	
<15	76 (67.9)
≥15	36 (32.1)
Menopause	
Yes	61 (54.5)
No	51 (45.5)
Number of pregnancies	
<4	75 (67.0)
≥4	37 (33.0)
Number of deliveries	
<3	95 (84.8)
≥3	17 (15.2)
Family history of cancer	
Yes	21 (18.8)
No	91 (81.2)

**Table 2:** Immunohistochemical reactivity of PR, ER, NF-*κ*B, and TNF-*α* (*n* = 112).

Parameters	Expression	Cancer tissue (%)	Normal tissue (%)	*χ* ^2^	*P* value
ER	+	85 (76)	17 (15)	83.23	<0.0001
	−	27 (24)	95 (85)		

PR	+	75 (67)	20 (18)	55.29	<0.0001
	−	37 (33)	92 (82)		

NF-*κ*B	+	94 (84)	0 (0)	162.0	<0.0001
	−	18 (16)	112 (100)		

TNF-*α*	+	105 (94)	2 (2)	189.8	<0.0001
	−	7 (6)	110 (98)		

PR: progesterone receptor; ER: estrogen receptor; NF-*κ*B: nuclear factor-*κ*B; TNF-*α*: tumor necrosis factor-*α*.

**Table 3:** The relationship between PR/ER and TNF-*α*/NF-*κ*B expression (*n* = 112).

Receptors		TNF-α expression		*χ* ^2^	*P* value	NF-*κ*B		*χ* ^2^	*P* value
		+	−			+	−		
PR	+	62	13	5.694	0.0170	74	1	36.56	<0.0001
	−	23	14			20	17		

ER	+	70	15	8.043	0.0046	80	5	27.14	<0.0001
	−	15	12			14	13		

PR: progesterone receptor; ER: estrogen receptor; NF-*κ*B: nuclear factor-*κ*B; TNF-*α*: tumor necrosis factor-*α*.

**Table 4:** The relationship between ER and PR expression as well as the relationship between NF-*κ*B and TNF-*α* expression (*n* = 112).

		PR (*n*)		*χ* ^2^	*P* value	NF-*κ*B (*n*)		*χ* ^2^	*P* value
		+	−			+	−		
ER (*n*)	+	73	12	57.04	<0.0001				
	−	2	25						

TNF-*α* (*n*)	+					90	15	3.972	0.046
	−					4	3		

PR: progesterone receptor; ER: estrogen receptor; NF-*κ*B: nuclear factor-*κ*B; TNF-*α*: tumor necrosis factor-*α*.

**Table 5:** The relationship between these four parameters and clinicopathological features (*n* = 112).

Parameters	PR (*n*)		*χ* ^2^	*P*	ER (*n*)		*χ* ^2^	*P*	TNF-*α* (*n*)		*χ* ^2^	*P*	NF-*κ*B (*n*)		*χ* ^2^	*P*
	+	−			+	−			+	−			+	−		
Age (y)																
<50	39	17	0.36	0.55	40	16	1.22	0.27	54	2	1.37	0.24	4	12	1.85	0.17
≥50	36	20			45	11			51	5			5	6		
Histologic subtype																
DC	65	32	3.77	0.44	76	21	5.22	0.27	91	6	3.13	0.54	8	14	2.18	0.70
ILC	3	3			4	2			6	0			4	2		
MA	2	2			2	2			3	1			3	1		
IC	4	0			3	1			4	0			3	1		
LC *in situ *	1	0			0	1			1	0			1	0		
TNM stages																
I-II	36	30	11.20	**<0.01**	45	21	7.57	**<0.01**	59	7	5.20	**0.02**	4	17	11.18	**<0.01**
III-IV	39	7			40	6			46	0			4	1		
Tumor grade																
I-II	39	36	22.98	**<0.01**	50	25	10.56	**<0.01**	68	7	3.68	0.06	5	17	7.32	**<0.01**
III	36	1			35	2			37	0			3	1		
Tumor size																
<5 cm	50	27	0.46	0.50	57	20	0.47	0.49	75	2	5.61	**0.01**	6	17	6.59	**0.01**
≥5 cm	25	10			28	7			30	5			3	1		
Lymph node metastasis																
Yes	50	10	15.65	**<0.01**	55	5	17.57	**<0.01**	60	0	8.62	**<0.01**		2	15.55	**<0.01**
No	25	27			30	22			45	7			3	16		
Age at menarche (y)																
<15	55	21	3.12	0.08	58	18	0.02	0.88	70	6	1.09	0.30	6	12	0.01	0.91
≥15	20	16			27	9			35	1			3	6		
Menopause																
Yes	44	17	1.62	0.20	45	16	0.33	0.57	55	6	2.94	0.09	5	10	0.01	0.92
No	31	20			40	11			50	1			4	8		
Number of pregnancies																
<4	50	25	0.01	0.92	60	15	2.09	0.15	70	5	0.07	0.80	6	11	0.33	0.56
≥4	25	12			25	12			35	2			3	7		
Number of deliveries																
<3	61	34	2.15	0.14	75	20	3.19	0.07	90	5	1.04	0.31	7	16	0.28	0.60
≥3	14	3			10	7			15	2			1	2		
Family history																
Yes	16	5	1.00	0.32	14	7	1.20	0.27	20	1	0.10	0.76	1	6	3.00	0.08
No	59	32			71	20			85	6			7	12		

PR: progesterone receptor; ER: estrogen receptor; NF-*κ*B: nuclear factor-*κ*B; TNF-*α*: tumor necrosis factor-*α*. PR: progesterone receptor; ER: estrogen receptor; NF-*κ*B: nuclear factor-*κ*B; TNF-*α*: tumor necrosis factor-*α*; DC: ductal carcinoma; ILC: invasive lobular carcinoma; MA: mucinous adenocarcinoma; IC: intraductal carcinoma; IC *in situ*: lobular carcinoma *in situ*.

## References

[1] Soerjomataram I, Lortet-Tieulent J, Parkin DM (2012). Global burden of cancer in 2008: a systematic analysis of disability-adjusted life-years in 12 world regions. *The Lancet*.

[2] Rakha EA, Reis-Filho JS, Ellis IO (2010). Combinatorial biomarker expression in breast cancer. *Breast Cancer Research and Treatment*.

[3] Early Breast Cancer Trialists Collaborative Group (2005). Effects of chemotherapy and hormonal therapy for early breast cancer on recurrence and 15-year survival: an overview of the randomized trials. *The Lancet*.

[4] Goldberg JE, Schwertfeger KL (2010). Proinflammatory cytokines in breast cancer: mechanisms of action and potential targets for therapeutics. *Current Drug Targets*.

[5] Chavey C, Bibeau F, Gourgou-Bourgade S (2007). Oestrogen receptor negative breast cancers exhibit high cytokine content. *Breast Cancer Research*.

[6] van der Burg B, van der Saag PT (1996). Nuclear factor-kappa-B/steroid hormone receptor interactions as a functional basis of anti-inflammatory action of steroids in reproductive organs. *Molecular Human Reproduction*.

[7] Goddard LM, Ton AN, Org T (2013). Selective suppression of endothelial cytokine production by progesterone receptor. *Vascular Pharmacology*.

[8] Brown CM, Mulcahey TA, Filipek NC, Wise PM (2010). Production of proinflammatory cytokines and chemokines during neuroinflammation: novel roles for estrogen receptors *α* and *β*. *Endocrinology*.

[9] Fromowitz FB, Viola MV, Chao S (1987). Ras p21 Expression in the progression of breast cancer. *Human Pathology*.

[10] Althuis MD, Fergenbaum JH, Garcia-Closas M, Brinton LA, Madigan MP, Sherman ME (2004). Etiology of hormone receptor-defined breast cancer: a systematic review of the literature. *Cancer Epidemiology Biomarkers and Prevention*.

[11] Payne SJL, Bowen RL, Jones JL, Wells CA (2008). Predictive markers in breast cancer: the present. *Histopathology*.

[12] Dowsett M, Houghton J, Iden C (2006). Benefit from adjuvant tamoxifen therapy in primary breast cancer patients according oestrogen receptor, progesterone receptor, EGF receptor and HER2 status. *Annals of Oncology*.

[13] Nadji M, Gomez-Fernandez C, Ganjei-Azar P, Morales AR (2005). Immunohistochemistry of estrogen and progesterone receptors reconsidered: experience with 5,993 breast cancers. *The American Journal of Clinical Pathology*.

[14] Liu S, Chia SK, Mehl E (2010). Progesterone receptor is a significant factor associated with clinical outcomes and effect of adjuvant tamoxifen therapy in breast cancer patients. *Breast Cancer Research and Treatment*.

[15] Deblois G, Giguère V (2013). Oestrogen-related receptors in breast cancer: control of cellular metabolism and beyond. *Nature Reviews Cancer*.

[16] Locksley RM, Killeen N, Lenardo MJ (2001). The TNF and TNF receptor superfamilies: integrating mammalian biology. *Cell*.

[17] Clarke RB, Howell A, Potten CS, Anderson E (1997). Dissociation between steroid receptor expression and cell proliferation in the human breast. *Cancer Research*.

[18] Berberoglu U, Yildirim E, Celen O (2004). Serum levels of tumor necrosis factor alpha correlate with response to neoadjuvant chemotherapy in locally advanced breast cancer. *International Journal of Biological Markers*.

[19] Cai D, Yuan M, Frantz DF (2005). Local and systemic insulin resistance resulting from hepatic activation of IKK-*β* and NF-*κ*B. *Nature Medicine*.

[20] Korkaya H, Liu S, Wicha MS (2011). Breast cancer stem cells, cytokine networks, and the tumor microenvironment. *Journal of Clinical Investigation*.

[21] Perrot-Applanat M, Vacher S, Toullec A (2011). Similar NF-*κ*B gene signatures in TNF-*α* treated human endothelial cells and breast tumor biopsies. *PLoS ONE*.

[22] Ben-Baruch A (2003). Host microenvironment in breast cancer development. Inflammatory cells, cytokines and chemokines in breast cancer progression: reciprocal tumor-microenvironment interactions. *Breast Cancer Research*.

[23] Yu M, Zhou X, Niu L (2013). Targeting transmembrane TNF-alpha suppresses breast cancer growth. *Cancer Research*.

[24] Kamali-Sarvestani E, Gharesi-Fard B, Sarvari J, Talei A-A (2005). Association of TNF-*α* and TNF-*β* gene polymorphism with steroid receptor expression in breast cancer patients. *Pathology and Oncology Research*.

[25] Purohit A, Newman SP, Reed MJ (2002). The role of cytokines in regulating estrogen synthesis: implications for the etiology of breast cancer. *Breast Cancer Research*.

[26] An J, Ribeiro RCJ, Webb P (1999). Estradiol repression of tumor necrosis factor-*α* transcription requires estrogen receptor activation function-2 and is enhanced by coactivators. *Proceedings of the National Academy of Sciences of the United States of America*.

[27] Panahi M, Saki N, Ashourzadeh S (2013). Expressional correlation of human epidermal growth factor receptor 2, estrogen/progesterone receptor and protein 53 in breast cancer. *Asian Pacific Journal of Cancer Prevention*.

[28] Almasri NM, Al Hamad M (2005). Immunohistochemical evaluation of human epidermal growth factor receptor 2 and estrogen and progesterone receptors in breast carcinoma in Jordan. *Breast Cancer Research*.

[29] Ashba J, Traish AM (1999). Estrogen and progesterone receptor concentrations and prevalence of tumor hormonal phenotypes in older breast cancer patients. *Cancer Detection and Prevention*.

[30] Clark GM, Osborne CK, McGuire WL (1984). Correlations between estrogen receptor, progesterone receptor, and patient characteristics in human breast cancer. *Journal of Clinical Oncology*.

